# LC-MS profiling and antioxidant, antifungal, and anticancer potentials of Tunisian *Allium sativum* L. extracts

**DOI:** 10.1371/journal.pone.0325227

**Published:** 2025-06-09

**Authors:** Ridha Ghali, Inès Limam, Ines Kassrani, Manel Araoud, Ezzedine Nouiri, Fatma Ben-Aissa Fennira, Mohamed Abdelkarim, Abderrazak Hedilli

**Affiliations:** 1 Toxicology and Environmental Research Laboratory, Centre Mahmoud Yaakoub of Urgent Medical Assistance, Tunis, Tunisia; 2 Department of Fundamental Sciences, Higher Institute of Biotechnology of Sidi Thabet, Manouba University, Manouba, Tunisia; 3 Human Genetics Laboratory, Faculty of Medicine of Tunis, Tunis El Manar University, Tunis, Tunisia; 4 Onco-Haematology Laboratory, Faculty of Medicine of Tunis, Tunis El Manar University, Tunis, Tunisia; University of Brescia: Universita degli Studi di Brescia, ITALY

## Abstract

Despite Garlic’s (*Allium. sativum*) long-standing reputation for therapeutic properties, comprehensive studies on Tunisian garlic are lacking. This study aims to evaluate different Tunisian *A. sativum* extracts rich in bioactive compounds (phenolic acids, flavonoids, and vitamins), exploring their potential bioactivities (antifungal, antioxidant, and cytotoxic). *A. sativum* samples underwent hexane, ethyl acetate, methanol, and water-based extractions. LC-MS quantification assessed bioactive compounds. Antioxidant activity was determined via the DPPH assay, antifungal effects were evaluated against *Aspergillus spp*., and cytotoxic effects were assessed using the MTT assay on *U266* human multiple myeloma and *MDA-MB-231* metastatic breast cancer cell lines. The aqueous extract exhibited the highest phenolic acid content (96.25 mg/kg fw) and the most water-soluble vitamins (14.69 mg/kg fw). In contrast, the methanol extract was richest in flavonoids, while the ethyl acetate extract had the highest concentration of fat-soluble vitamins (20.21 mg/kg fw). Both aqueous and methanolic extracts demonstrated potent antioxidant activity. The aqueous extract exhibited the strongest antifungal activity (MIC: 1.5 mg/mL for *A. flavus* and 3 mg/mL for *A. niger*). Furthermore, the ethyl acetate extract showed remarkable cytotoxic effects against cancer cell lines, indicating its potential as an effective agent against metastatic breast cancer and refractory multiple myeloma. *A. sativum* emerges as a functional food source with antioxidant, antifungal, and cytotoxic activities, particularly against multiple myeloma. While this study provides a strong foundation for further exploration, additional research is needed to identify active compounds, elucidate mechanisms, and assess therapeutic potential.

## Introduction

The potential of plant-based foods as both preventive and therapeutic tools in combating diseases such as cardiovascular disease and cancer is a burgeoning field of research, supported by promising evidence [1]. Numerous studies suggest a robust association between a diet rich in fruits, vegetables, and legumes and a reduced risk of developing various cancers, including colorectal, breast, prostate, and epithelial ovarian cancer [2]. This protective effect is attributed to the synergistic interplay of bioactive compounds in these foods. Vitamins, essential for maintaining body homeostasis, have been proposed as part of preventive strategies against cancer, although the results remain heterogeneous [3]. Specifically, vitamin deficiencies have been identified as cofactors in oncogenesis [3,4]. Additionally, antioxidants such as polyphenols have demonstrated the ability to inhibit cancer cell growth and promote apoptosis [5–7]. Furthermore, emerging research investigates the integration of certain medicinal plants, particularly those from the Allium genus, into cancer treatment regimens due to their observed ability to enhance the efficacy of conventional therapies and mitigate side effects [8]. While still in its early stages, this field holds great promise for future cancer prevention and treatment strategies, empowering individuals to leverage the nutritional power of their diet as a valuable tool against this intricate disease.

*Allium sativum L.* (*A. sativum)*, a member of the Amaryllidaceae family, is traditionally cultivated from individual cloves planted in the fall. Diverse varieties exhibit notable differences in taste, size, and bulb color [9–11]. Rich in historical significance, garlic has been documented in ancient civilizations, including Egyptian, Greek, and Roman cultures, and continues to play a prominent role in various cultural practices worldwide [12]. With its pivotal role in human diets and widespread use in traditional medicine, numerous studies have highlighted garlic’s wealth of biologically active compounds, including flavonoids and sulfur compounds like alliin (S-allyl cysteine sulfoxide) and its derivatives [12–15]. These compounds are responsible for garlic’s well-documented antiseptic, antibacterial, antiparasitic, bactericidal, and bacteriostatic activities [10,11,16]. Furthermore, experimental and clinical studies have demonstrated garlic’s beneficial effects on cardiovascular health, including antihypertensive properties, inhibition of thrombosis, platelet aggregation, reduction in plasma viscosity, unstable angina, and peripheral arterial occlusive disorders and improvements in blood vessel elasticity and capillary perfusion [17–19].

Many previous studies highlight the potential health benefits of garlic, particularly its strong antioxidant properties [20,21]. These effects are largely attributed to sulfur-containing compounds, such as allicin and diallyl di- and tri-sulfides [21–23]. Furthermore, bioactive components like vitamins, selenium, and polyphenols found in Allium species contribute also to their antioxidant activity [24,25]. Numerous studies have also documented the therapeutic potential of *A. sativum*, especially about gastric, intestinal, and colorectal cancers [26,27]. Notably, Alliin, a precursor of allicin in garlic extract, has been shown to inhibit the proliferation of gastric adenocarcinoma cells by modulating apoptosis [26].

In Tunisian traditional medicine and cuisine, garlic, known as “thoum,” has long been recognized for its culinary and potential medicinal attributes. Despite its widespread use, our bibliographic research revealed a lack of comprehensive studies on the composition of this culinary mainstay. To address this gap, our research team conducted a thorough physicochemical analysis using liquid chromatography-mass spectrometry (LC-MS) to elucidate the detailed composition of Tunisian garlic, specifically focusing on its polyphenol content, including phenolic acids and flavonoids, as well as certain water-and fat-soluble vitamins. This was achieved through successive extractions using solvents of increasing polarity, such as hexane, ethyl acetate, and methanol. Additionally, a crude aqueous garlic extract was analyzed in parallel to compare differences in polyphenol composition. Furthermore, we evaluated specific pharmacological effects, including antioxidant activity, antifungal properties against two fungi, and potential anticancer effects using two resistant cell lines: *U266* human multiple myeloma cells and *MDA-MB-231* cells, representing an aggressive triple-negative breast cancer. Through solvent extractions, this study focused on quantifying polyphenols and specific vitamins in Tunisian garlic. The ethyl acetate extract demonstrated notable activity, highlighting its potential for further exploration. These preliminary findings provide a basis for future research on garlic’s therapeutic applications.

## Materials and methods

### Sample procurement and extracts preparation

About 100 *A. sativum* adult plants were obtained from a local farmer in Ksar Mezouar (36°46'60" N et 9°19'60" E), Beja Governorate, in North Tunisia in early May. In Tunisia, no permits are required to conduct scientific research on phyto resources within the country. However, permission is mandatory if samples need to be sent abroad. The plant materials were identified based on Tunisian flora literature and morphological characteristics of leaves and bulbs described in descriptors for Allium spp. [9,28,29]. The garlic bulb was washed, peeled, and crushed using a mortar. Bioactive compounds were then extracted via maceration using solvents of varying polarities (water, methanol, ethyl acetate, and hexane), following the methodology outlined by Shetty [30]. Approximately 100 g of crushed garlic was extracted using 400 mL of each solvent using an automatic shaker for 24 hours at room temperature. This approach was designed to selectively isolate compounds based on their differential solubilities in solvents.

The mixture was then filtered using a Buchner funnel, followed by centrifugation for 10 min at 3000 rpm. The upper phase was then evaporated to dryness using a rotary evaporator (Heidolph Instruments, Schwabach, Germany). The resulting dry residues were reconstituted in methanol and adjusted to a concentration of 40 mg/mL. The aqueous extract macerate was filtered through filter paper, dried in SpeedVac vacuum concentrators SPD1030 (Thermo Scientific, Lissieu, France), and stored at +4°C until use. All samples were processed in triplicate.

### Chemicals and standard solutions

Extraction solvents: methanol, ethyl acetate, and hexane were of analytical grade, and LC-MS-grade methanol, formic, and acetic acids were obtained from VWR International (Rosny-sous-Bois, France). Analytical standard solutions of phenolic acids, flavonoids, and vitamins (purity ≥ 97%), 2,2-diphenyl-1-picrylhydrazyl (DPPH), and DMSO were purchased from Sigma Chemical Co. (St. Louis, MO, USA). Purified water was obtained using a Multi Q system from Millipore (Darmstadt, Germany).

### Fungal strains and human cancer cell origins and culture conditions

The fungal strains tested for antifungal activities were *Aspergillus flavus* strain SRRC 1000A and *Aspergillus niger* strain CBS 513, hereafter referred to as *A. flavus* and *A. niger*, respectively. These strains were previously isolated from food samples, identified using conventional methods and ITS1-5.8S-ITS2 region sequencing of the nuclear ribosomal DNA, and stored in the laboratory of Toxicology, Centre Mahmoud Yaakoub of Urgent Medical Assistance, Tunis, Tunisia. Antifungal activities were investigated using a Dichloran Rose Bengal Chloramphenicol (DRBC) medium (Scharlau, Barcelona, Spain).

To study the extracts’ anticancer activities, two cell lines were used: *U266* human multiple myeloma cells, generously provided by Professor Brigitte Sola [31], and *MDA-MB-231*, a human breast cancer cell line purchased from ATCC (ATCC HTB-26) [32]. Both cell lines were maintained under standard culture conditions (at 37 °C and 5% CO_2_) using RPMI 1640 and DMEM media, respectively. Cell culture media were supplemented with 10% fetal bovine serum, 2 mM L-glutamine, and penicillin 0.1 μg/μL/streptomycin 0.1 μg/μL solution, obtained from PAN-Biotech (Aidenbach, Germany).

### LC-MS analysis

The quantitative analysis of phenolic acids, flavonoids, water-and fat-soluble vitamins across diverse fractions of *A. sativum* was conducted employing an 8020 LC-MS system including an LC-20AD XR binary pump system, SIL-20 AC XR autosampler, CTO-20 AC column oven and quadrupole mass spectrometer equipped with an electrospray ionization (ESI) and atmospheric pressure chemical ionization (APCI) modules and piloted by LabSolutions software (Shimadzu, Kyoto, Japan). Previously validated methods were applied [33]. Chromatograms and mass spectra for single-component standard solutions were employed for analyte identification, based on their relative retention times and specific molecular ions. Calibration curves were established and validated prior to sample analysis using freshly prepared calibration solutions and were employed for quantitative determination. The biomolecule contents in the analyzed extracts are reported as mean values ± standard deviation in mg/g of garlic fresh weight (fw).

#### Phenolic compound LC-MS identification and quantification.

The quantification of 15 phenolic acids and 15 flavonoids was carried out on *A. sativum* extracts. A reverse-phase C18 column (100 x 2.1 mm x 3 µm), maintained at a consistent temperature of 40°C, was used for chromatographic separation at a 0.4 mL/min flow rate. The mobile phase consisted of an aqueous solution of 20 mmol ammonium formate and 0.1% formic acid (A) and a solution of 0.1% formic acid in methanol (B). The separation was performed using an elution gradient optimized as follows: 10% −100% B for 45 min., 100% B for 45−50 min., and return to the initial condition (10% B) over 5 min. The mass spectrometer operated under the following conditions: Nitrogen served as the nebulizing gas at a flow rate of 1.2 L/min, with a drying line temperature maintained at 250°C. The capillary voltage was set at −3.5 V, while the detector voltage was set at 1.1 V. Mass spectra were collected in negative ion mode across a mass-to-charge ratio (m/z) range of 50–1200. The sought molecular ions used for SIM are described in Table 1.

**Table 1:** Retention time and sought molecular Ions (M/Z) used for SIM of explored polyphenolic compounds.

Phenolic acids	MZ	RT (min)	Flavonoids	MZ	RT (min)
Caffeic acid	179.0	12.72	Acacetin	283.6	35.95
Chlorogenic acid	353.0	11.88	Apigenin	269.5	30.02
o – Coumaric acid	163.0	18.71	Apigenin-7-O-glucoside	431.9	22.36
p – Coumaric acid	163.0	16.02	Catechin	289.6	9.51
Gallic acid	169.0	4.83	Cirsilineol	343.0	32.34
Protocatechuic acid	153.0	7.37	Cirsiliol	329.0	30.33
Quinic acid	191.0	2.37	Epicathechin	289.0	12.44
Rosmarinic acid	359.0	19.92	Luteolin	285.6	27.87
Salvianolic acid A	717.0	20.70	Luteolin-7-O-glucoside	447.9	20.75
Syringic acid	197.0	13.59	Naringenin	271.5	24.40
Trans-Cinnamic acid	147.0	23.14	Naringin	579.0	18.90
Trans-Ferulic acid	193.0	17.30	Quercetin	447.8	22.64
4-O-caffeoylquinic acid	353.0	13.00	Hyperoside	463.9	20.60
1,3-di-O-caffeoylquinic acid	515.0	13.36	Rutin	609.1	20.58
4,5-di-O-caffeoylquinic acid	515.0	22.61	Silymarin	482.0	25.49

RT: retention times. MZ: molecular ions collected in negative ion mode.

#### Water-soluble vitamins LC-MS identification and quantification.

Chromatographic separation of nine water-soluble vitamins was executed utilizing a mobile phase composed of an aqueous mixture of 20 mmol ammonium formate and 0.1% aqueous formic acid (A) and 0.1% formic acid in methanol (B). The linear gradient profile (A: B) began with 2% B and was maintained over 2 min. Subsequently, it linearly decreased to 55% B for 13 min, followed by a 2-minute hold at 55% B. Finally, it further decreased to 100% B during the next 3 min. The flow rate was set to 0.4 mL/min, with a total runtime of 20 min. The column temperature was maintained at 40°C, and the injection volume was 5 µL.

The mass detector was operated in negative ion conditions in the SIM mode, scanning the range of 50–1200 m/z. The specific molecular ions (m/z) applied for SIM are summarized in Table 2. The ESI source parameters were a capillary voltage of −3.5 V, and a detector voltage of 1.2 V, desolvatation line and heat block temperatures of 250°C and 500°C, respectively, a nebulizing gas flow of 1.5 L/min, and a drying gas flow of 15 L/min.

**Table 2:** Explored water- and fat-soluble vitamins.

Water-soluble vitamins			Fat-soluble vitamins		
Vitamin	M/Z	RT	Vitamins	M/Z	RT
**B1** (Thiamine)	265	4.78	**A** (Retinol)	269.2	12.68
**B2** (Riboflavin)	377	14.17	**D2** (Ergocalciferol)	397.3	16.95
**B3** (Niacine)	124	4.32	**D3** (Cholecalciferol)	385.4	16.76
**B5** (Pantothenic acid)	220	8.60	**K1** (Phylloquinone)	451.4	20.79
**B6** (Pyridoxine)	170	5.78	**K2** (Menaquinone)	445.3	17.79
**B7** (Biotin)	245	13.01	**E** (Alpha tocopherol)	431.3	18.36
**C** (Ascobic acid)	175	2.42	**ProVitamin A** (Beta carotene)	537.5	26.84

RT: retention times. MZ: molecular ions collected in negative ion mode for water- soluble vitamins and in negative ion mode for fat-soluble vitamins.

#### Fat-soluble vitamins LC-MS identification and quantification.

Fat-soluble vitamins were quantified exclusively in the hexane and ethyl acetate extracts. Vitamins A, D, E, and K are predominantly hydrophobic, insoluble in water, and more soluble in nonpolar organic solvents. Chromatographic separation was performed using an Aquasil C18 column (150 mm × 3 mm, 3 µm) from Thermo Electron (Dreieich, Germany). The mobile phase consisted of A (5 mmol ammonium formate + 0.1% aqueous formic acid) and B (0.1% formic acid in methanol), with a linear gradient elution from 70% to 100% B over 15 min, followed by 100% B from 15 to 45 min, at a flow rate of 0.4 mL/min. The column temperature was maintained at 40°C, and the injection volume was 5 µL.

Mass spectrometric detection was performed in SIM mode across a range of 50–1200 m/z using atmospheric pressure chemical ionization (APCI) in negative ionization mode. Nitrogen was used as nebulizing gas at a flow rate of 2 L/min, with auxiliary dry gas set at 5 L/min. The dissolving line temperature was 250°C, and the block source and the APCI temperatures were both set to 400°C. The detector voltage was maintained at 1.2 V, while the capillary voltage was set to −3.5 V. The specific molecular ions used for SIM are summarized in Table 2.

### Determination of antioxidant activity

The 2,2-diphenyl-1-picrylhydrazyl (DPPH) free radical scavenging assay was employed to evaluate the antioxidant potential, as described by Kirby and Schmidt [34]. Briefly, 1 mL of extracts at variable concentrations (2.5, 5, 10, 20, and 40 mg/mL) was added to 1 mL of a DPPH solution (0.2 mM in ethanol) as the free radical source and incubated for 30 min at room temperature. The reduction of DPPH by the samples was measured at 517 nm using a PG Instruments T60 spectrophotometer (Lutterworth, UK). The scavenging activity was calculated as a percentage of inhibition (PI) by comparing it to the negative control and using the formula: DPPH radical scavenging activity (%) = [(A0−A1)/A0×100], where *A*_0_ represents the absorbance of the control and *A*_1_ represents the absorbance of the test sample. L-ascorbic acid was used as the positive control, the IC₅₀ was evaluated for each extract and the assay was performed in triplicate.

### Determination of antifungal activities

With minor adjustments, the disc diffusion method, as reported by Jorgensen and Turnidge [35], was employed to evaluate the antifungal activity of *A. sativum* extracts. Briefly, a paper disk was loaded with 15 µL of various extract concentrations (2.5, 5, 10, 20, and 40 mg/mL) in 2% DMSO before being placed on agar plates seeded with the tested fungal strain. Paper discs soaked with 2% DMSO alone were included as controls in each plate. Plates were then incubated for 48–72 h at 25°C. Negative control plates contained disks without plant extract, and other plates with disks saturated with 15 μl of carbendazim solution (10 mg/mL) served as a positive control was also prepared and incubated.

Antifungal activity was assessed by measuring the inhibition-zone diameter around the disks in cm, with each experiment conducted in triplicate. Antifungal efficacy was presented as the mean inhibition zone (cm) ± standard deviation. The minimum inhibitory concentration (MIC) of the studied extracts was determined through broth macro-dilution in 1 mL standard test tubes, following the method described by Arendrup and colleagues with slight modifications [36,37].

### Determination of cytotoxicity activities

Cell viability was investigated using the 3-(4,5-dimethylthiazol-2-yl)-2,5-diphenyltetrazolium bromide (MTT) colorimetric assay obtained from Sigma-Aldrich, following a validated protocol [38]. In brief, *U266* and *MDA-MB-231* cells were exposed to varying concentrations (15.56, 31.125, 62.25, 125, 250, and 500 μg/mL) of distinct solvent extracts for either 24 or 48 h. At the end of the experiment, 20 μL of MTT solution (5 mg/mL) was added to each well, and the plates were incubated for 4 h at 37 °C. The resultant purple/blue MTT formazan precipitate was dissolved in 100 μL of 10% DMSO. Absorbance was measured at 490 nm using an ELISA plate reader (Model EXL800; BioTek, USA). The growth inhibition percentage was calculated using the formula: % Inhibition=100 – ((treated OD/non−treated OD) * 100). Experiments were performed in triplicate, and all values are presented as mean ± SD. The obtained results were also used to determine the IC50 values.

### Statistical analysis

Each experiment was conducted with three independent analyses to ensure the robustness and reliability of the results. Statistical analysis was conducted using SPSS version 20 and GraphPad Prism 5 software (for MTT assay). From this analysis, mean values and standard deviations (SD) were calculated. Statistical significance between the results was assessed using the student’s t-test with a probability threshold of p < 0.05, and correlation studies between the biomolecule levels and the antioxidant, antifungal, and antiproliferative activities were tested based on Pearson’s and Spearman’s correlation coefficients.

## Results and discussion

### Yield indices from various extractions

Following the application of various individual solvent-based extraction methods, the total yield rate of each extract was determined. The calculation results revealed that the aqueous extracts exhibited the highest yield rate (5.80 ± 3.11%). Similarly, the yield rate of the methanolic extract was notably high, reaching 5.31 ± 2.24%. Conversely, ethyl acetate and hexane extracts displayed the lowest extraction rates, 0.42 ± 0.31% and 0.14 ± 0.08%, respectively. Aqueous and methanolic extracts demonstrated substantially higher yield rates compared to ethyl acetate and hexane extracts. This suggests that the choice of solvent significantly impacts the efficiency of the extraction process, potentially influencing the overall composition and bioactivity of the obtained extracts. The selection of hexane, ethyl acetate, methanol, and water as extraction solvents is guided by their polarity and affinity for specific bioactive compounds in *A. sativum.* Each solvent targets compounds with varying solubilities. Water and methanol efficiently extract hydrophilic and polar compounds like polyphenols and vitamins, yielding higher amounts, while ethyl acetate and hexane target moderately polar and lipophilic compounds (e.g., flavonoids, terpenoids, and fat-soluble vitamins) with lower yields. The Solvent choice is critical for optimizing extraction efficiency and composition [39–41].

### Phenolic acid content in samples

Table 3 presents the LC-MS analysis of sixteen phenolic acids in garlic samples extracted using four different solvents. The varied phenolic acid content across extracts can be attributed to compound responses to distinct extraction conditions. Quantitatively, the aqueous extract exhibited the highest content (96.25 mg/kg fw), followed by methanolic and ethyl acetate extracts (48.10 and 10.36 mg/kg fw), while hexane extract had the lowest content (0.82 mg/kg fw). Significant differences (p < 0.05) were observed between all extract types. The aqueous extract is the most promising for applications requiring high polyphenol content, such as those leveraging antioxidant properties. Previous studies conducted by Chakki and collaborators on Tunisian *A. sativum* collected in autumn from a different region revealed interesting findings regarding phenolic content. Results showed that the 70% methanolic extract contained a total phenolic content of 10.6 mg GA/g, while the 80% methanolic extract, within 72 h of incubation, yielded 5 mg GA/g [22]. It is well known that the composition of garlic bulbs is strongly influenced by soil characteristics, broad climatic conditions, and weather variations. Additionally, differences in experimental protocols and conditions may further explain the observed discrepancies [42].

**Table 3:** Phenolic acids contents (mg/kg fw) in the various *A. sativum* extracts.

Phenolic acids	Hexane Extract	Ethyl acetate extract	Methanolic extract	Aqueous extract
Caffeic acid	ND	0.63 ± 0.13	2.33 ± 0.39	ND
Chlorogenic acid	ND	ND	ND	ND/
o – Coumaric acid	ND	ND	ND	ND
p – Coumaric acid	ND	1.90 ± 0.32	5.54 ± 0.92	2.9 ± 0.48
Gallic acid	ND	ND	ND	ND
Protocatechuic acid	ND	ND	ND	ND
Quinic acid	0.56 ± 0.08	ND	16.52 ± 3.11	ND
Rosmarinic acid	ND	ND	0.37 ± 0.09	ND
Salvionolic acid A	0.26 ± 0.03	ND	ND	0.99 ± 0.09
Syringic acid	ND	ND	17.27 ± 3.22	ND
Trans-Cinnamic acid	ND	ND	ND	ND
Trans-Ferulic acid	ND	7.52 ± 1.82	ND	92.18 ± 7.32
4-o-caffeoylquinic acid	ND	ND	5.88 ± 1.11	ND
1,3-Dicaffeoylquinic acid	ND	ND	0.20 ± 0.02	0.17 ± 0.03
4,5-Dicaffeoylquinic acid	ND	0.31 ± 0.07	ND	ND
Total concentration	0.82 ± 0.11	10.36 ± 2.33	48.10 ± 8.76	96.25 ± 7.92

ND: Not detected, Values in columns are the average of three analyses ± SD.

Qualitative analysis demonstrated that the methanol extraction recovered seven out of the sixteen phenolic acids. Water and ethyl acetate extraction were also effective, retrieving four compounds each. Notably, trans-ferulic acid was the most abundant phenolic acid, found in aqueous and ethyl acetate extracts (92.18 and 7.52 mg/kg fw, respectively), followed by syringic acid, exclusively detected in the methanol extract (17.27 mg/kg fw). Interestingly, syringic acid and p-hydroxybenzoic acid derivatives were most abundant in the methanolic extract, followed in descending order by ethanol, acetone, and hexane during the extraction process from two Egyptian garlic varieties [43]. Quinic acid (16.52 mg/kg fw in methanol extract) and its derivatives were identified, with varying concentrations in methanol, aqueous, and ethyl acetate extracts. However, according to the literature, quinic acid has rarely been reported in studies on garlic bulbs, where another metabolite, chlorogenic acid, is more commonly detected. For instance, Nagella et al. identified chlorogenic acid in three out of five aqueous extracts of garlic cultivated in different locations in Korea [44]. Interestingly, Chahbani and colleagues reported that quinic acid was the predominant phenolic acid in all dried samples (772.02–1026.56 µg/g extract) from Tunisian garlic leaves [45]. Moreover, p-coumaric acid was also identified in methanol, water, and ethyl acetate extracts, with concentrations of 5.54, 2.9, and 1.9 mg/kg fw, respectively. According to Szychowski and collaborators, the analysis of nine aqueous extracts from raw garlic grown in diverse regions-including Poland, Spain, China, Portugal, Burma, Thailand, and Uzbekistan- revealed the presence of p-coumaric acid in all cultivars, with concentrations ranging from 1 to 3.03 μg/g of raw garlic [46]. Contrary to our findings, where gallic acid was not detected, their study reported the presence of this compound in all nine garlic varieties. Methanol proved particularly effective for extracting caffeic acid (2.33 mg/kg fw compared to 0.63 mg/kg fw in ethyl acetate). For instance, Nagella et al. identified this compound in five aqueous extracts of Korean garlic [44]. Additionally, caffeic acid has been described as one of the major phenolic acids in ten Spanish garlic varieties from different regions, with an average concentration of 2.9 mg/kg [47].

### Flavonoid content in extracts

The LC-MS analysis of flavonoids in the four types of *A. sativum* extracts was conducted and the outcomes are displayed in Table 4. Methanol proved to be the most efficient solvent, exhibiting the highest recovered flavonoid concentration (424.87 mg/kg fw).

**Table 4:** Flavonoids contents (mg/kg fw) in the various *A. sativum* extracts.

Flavonoids	Hexane extract	Ethyl acetate extract	Methanolic extract	Aqueous extract
Acacetin	ND	ND	ND	ND
Apigenin	ND	ND	0.14 ± 0.03	ND
Apigenin-7-o-glucoside	ND	0.53 ± 0.07	1.11 ± 0.18	ND
Catechin	ND	ND	ND	ND
Cirsilineol	ND	5.30 ± 0.61	3.26 ± 0.42	ND
Cirsiliol	0.78 ± 0.12	10.63 ± 1.83	417.26 ± 30.40	77.70 ± 8.84
Epicathechin	ND	ND	ND	ND
Luteolin	ND	0.04 ± 0.01	1.69 ± 0.21	0.89 ± 0.14
Luteolin-7-O-glucoside	ND	ND	ND	ND
Naringenin	ND	ND	ND	ND
Naringin	ND	ND	ND	ND
Hyperoside	ND	ND	ND	ND
Quercetin	ND	0.47 ± 0.32	ND	ND
Rutin	ND	ND	1.41 ± 0.19	ND
Silymarin	ND	ND	ND	ND
Total concentration	0.78 ± 0.12	16.97 ± 2.83	424.87 ± 31.42	78.59 ± 8.98

ND: Not detected. Values in columns are the average of three analyses ± SD.

The quantity of flavonoids detected in this extract was nearly nine times higher than the phenolic content, resulting in a total polyphenol content of 473 mg/kg fw. This finding establishes methanol as the most effective solvent for polyphenol extraction, followed by the aqueous extract, which yielded approximately 175 mg/kg fw. These results are consistent with literature [44,48], such as the findings reported by Chakki and colleagues, who identified 59.6 QE mg/100 g of flavonoids in the 70% methanolic extract of Tunisian garlic (compared to 10.6 GAE mg/100 g of phenolic content) versus 13.2 QE mg/100 g in the ethanolic extract (compared to 43.6 mg GAE/100g of phenolic content). Similarly, Elhafez’s work highlighted methanol’s superiority in extracting the highest total polyphenol content, estimated at 402.64 and 383.90 µg/g for two Egyptian garlic varieties, outperforming both acetone and hexane, with the latter showing the weakest extraction efficiency. [43]. In contrast, Sergiova et al. reported significantly higher total phenolic content values, ranging from 797.11 to 1183.98 mg GAE/kg dm (dry material), and total flavonoid content ranging from 15.96 to 28.18 mg CE/kg dm [49]. Interestingly, a recent Tunisian study analyzing garlic leaves from southern Tunisia via LC–ESI–MS revealed flavonoids as the dominant category among identified compounds, representing 63% in fresh samples and up to 89.40% in samples dried at 40 °C [45]. In our study, Cirsiliol was identified as the predominant flavonoid in all four extracts, with concentrations of 417.26, 77.7, 10.63, and 0.78 mg/kg fw, for methanolic, aqueous, ethyl acetate and hexane extracts, respectively. Notably, this compound was also identified in Tunisian garlic leaves in recent work by Chahbani et al., further supporting our findings [45].

Qualitatively, the methanolic extract recovered the highest number of flavonoid compounds (six), followed by the ethyl acetate extract (five). These two extracts notably facilitated the identification of cirsilineol and apigenin-7-O-glucoside. Although luteolin was detected in methanolic, aqueous, and ethyl acetate extracts, its concentration remained relatively low (1.69 mg/kg fw vs. 0.89 and 0.04 mg/kg fw, respectively). Additionally, quercetin was exclusively detected in trace amounts in the ethyl acetate extract, whereas rutin and apigenin were detected in the methanolic extract. The flavonoids detected in this study were also identified in the leaves of Tunisian garlic, albeit in varying amounts, as reported by Chahbani et al. [45]. Such differences may be attributed to geographical variations, climate, and harvest season. Moreover, some flavonoids observed in other studies were not detected in our extracts. For instance, Elhafez et al. reported catechin and epicatechin as prominent flavonoids in methanolic extracts of Egyptian garlic [43]. These discrepancies could stem from differences in genetic makeup, environmental conditions (notably climatic variations and drought observed in recent years), solvent choice, and extraction methods [42,45]. Additionally, factors such as plant maturity at harvest and post-harvest handling likely influenced the flavonoid profile, contributing to the observed variations [42,50].

### Water-soluble vitamins content

The results of water-soluble vitamin quantification in extracts from different fractions of *A. sativum* revealed significant variations as presented in Table 5. In terms of total vitamins, extracts showed overall similar concentrations, slightly higher in methanolic and aqueous extracts (36.42 mg/ Kg), emphasizing the diversity of vitamin profiles in garlic extracts. Among the scrutinized vitamins, ascorbic acid and pyridoxine were the most relevant in all extracts. Vitamin C concentrations were high in methanolic and aqueous extracts, while hexane and ethyl acetate extracts showed lower concentrations. Similarly, pyridoxine concentrations were significantly higher in ethyl acetate, and aqueous extracts compared to hexane and methanolic extracts. In contrast, thiamine was not detected in any extracts, and nicotinamide was measurable at the lowest levels. These results align with previous work, which reported higher concentrations of vitamin C, ranging from 9.7 to 15.6 mg/100 g fw, followed by B vitamins, with vitamin B6 being the most abundant specifically in Italian *A. sativum* cultivar (2.04 mg/100 g fw) [51].

**Table 5:** Water-soluble vitamins contents (mg/kg fw) in the various *A. sativum* extracts.

Water soluble vitamins	Hexane extract	Ethyl acetate extract	Methanolic extract	Aqueous extract
Thiamine	ND	ND	ND	ND
Riboflavin	0.37 ± 0.05	0.39 ± 0.09	1.28 ± 0.18	1.26 ± 0.17
Nicotinamide	0.16 ± 0.02	ND	0.40 ± 0.07	ND
D-Pantothenic Acid	1.30 ± 0.19	0.83 ± 0.14	ND	ND
Pyridoxine	1.41 ± 0.24	2.23 ± 0.38	0.28 ± 0.04	2.44 ± 0.38
Biotin	0.66 ± 0.10	0.51 ± 0.08	1.14 ± 0.16	1.19 ± 0.16
Ascorbic acid	11.16 ± 1.976	11.13 ± 1.911	33.76 ± 3.981	32.79 ± 3.9
Total concentration	14.69 ± 2.69	14.70 ± 2.41	35.58 ± 4.45	36.42 ± 4.61

ND: Not detected. Values in columns are the average of three analyses ± SD.

The results of the vitamin concentration analysis in different extracts of *A. sativum* present intriguing insights into the nutritional composition and potential health benefits of garlic. The variations in vitamin content across different extraction methods highlight the importance of selecting the appropriate extraction solvent to maximize the recovery of specific vitamins. For instance, methanolic extraction appears particularly efficient in extracting thiamine and pantothenic acid, while riboflavin concentrations are notably higher in hexane and ethyl acetate extracts. The presence of vitamins such as pyridoxine and ascorbic acid across various extracts underscores garlic’s potential as a source of these essential nutrients [52,53].

These findings have implications for dietary supplementation and functional food development, as different extraction methods can yield garlic extracts with diverse vitamin profiles, tailored to specific nutritional needs and health objectives. Furthermore, understanding the variations in vitamin content among different extracts can guide the development of optimized extraction protocols to enhance the nutritional value of garlic-based products. Further research exploring the bioavailability and biological activities of these vitamins from garlic extracts would provide valuable insights into their potential health-promoting effects and therapeutic applications.

### Fat-soluble vitamins content

Table 6 displays the fat-soluble vitamin contents of *A. sativum* hexane and ethyl acetate extracts. Of the seven vitamins examined, only vitamins D3 and K3 were absent from both extracts. Compared to the hexane extract (2.85 ± 0.68 mg/kg fw), the ethyl acetate extract had the highest overall vitamin content (20.21 ± 4.01 mg/kg fw). Riboflavin (vitamin D2) was the most concentrated vitamin in both extracts, with an average concentration of 19.05 mg/kg fw for the ethyl acetate extract and 2.14 mg/kg fw for the hexane extract. Similar results have been reported by Okoro and associates, who found 0.05 mg riboflavin, 0.24 mg thiamine, 10 mg ascorbic acid, and 0.4 mg niacin in 100 g of garlic bulbs [10]. Another study quantified thiamine, riboflavin, and niacin in four *A. Sativum* cultivars in Italy from the Viterbo and Alvito areas in Italy, with respective mean concentrations of 0.26, 0.02, and 0.84 mg/100 g fw [51].

**Table 6:** Fat-soluble vitamins contents (mg/kg fw) in the various *A. sativum* extracts.

Fat-soluble vitamins	Hexane extract	Ethyl acetate extract
A (Retinol)	0.21 ± 0.05	0.22 ± 0.06
D2 (riboflavin)	2.14 ± 0.52	19.05 ± 3.85
D3 (Cholecalciferol)	ND	ND
K1 (d-Pantothenic acid)	0.50 ± 0.11	0.44 ± 0.13
K2 (Menaquinone)	ND	ND
E (Alpha tocopherol)	ND	0.50 ± 0.15
Pro-Vitamin A (Beta carotene)	0.026 ± 0.01	0.027 ± 0.01
Total concentration	2.85 ± 0.68	20.21 ± 4.01

ND: Not detected. Values in columns are the average of three analyses ± SD.

### Antioxidant potential of *A. sativum* extracts

The antioxidant effect of *A. sativum* extracts was evaluated using DPPH radical scavenging assay. Table 7 illustrates the percent inhibition (PI) of DPPH radical scavenging activity at different extracts concentrations.

**Table 7:** Percentage inhibition of DPPH scavenging activity and IC₅₀ values of the *A. sativum* extracts.

Extract concentrations (mg/mL)	Hexane extract	Ethyl acetate extract	Methanolic extract	Aqueous extract
	**Percentage inhibition of DPPH scavenging activity (%)**			
40	27.1 ± 8.9	39.3 ± 1.8	50.3 ± 5.3	75.1 ± 6.4
20	18.5 ± 6.3	23.6 ± 1.9	33.8 ± 4.9	48.7 ± 6.8
10	11.4 ± 4.9	16.1 ± 2.1	19.8 ± 3.4	29.5 ± 5.2
5	7.2 ± 3.2	9.8 ± 3.0	12.9 ± 3.1	17.4 ± 5.4
2.5	3.1 ± 2.8	4.5 ± 2.1	5.2 ± 1.9	7.8 ± 3.1
	**The Median Inhibitory Concentration, IC**₅₀ **(mg/mL)**			
**IC**₅₀ **(mg/mL)**	75.26	51.08	37.70	23.75

Values in columns are the average of three analyses ± SD.

Overall, these findings underscore the potent antioxidant activity of *A. sativum* extracts, with the aqueous extract exhibiting the highest antioxidant potential, followed by the methanolic, ethyl acetate, and hexane extracts, respectively. The median inhibitory concentrations (IC50) of extracts obtained using different solvents show significant variations in terms of efficacy. The hexane extract exhibits the lowest inhibitory activity, with an IC50 of 75.26 mg/mL, indicating a lower ability to inhibit the tested target. In contrast, the ethyl acetate extract demonstrates better efficacy, with an IC50 of 51.08 mg/mL. The methanolic extract and the aqueous extract prove to be even more effective, with IC50 values of 37.70 mg/mL and 23.75 mg/mL, respectively, the latter being the most active among all evaluated extracts.

These results align with previous studies highlighting the antioxidant potential of garlic extracts. For instance, Wani and Basir attributed superior antioxidant activity to methanolic extracts of Indian garlic, reporting 51% PI, compared to ethanolic (45%) and aqueous extracts (24%) [54]. Similarly, Awan et al. found that methanolic extracts of Pakistani garlic exhibited a PI of 61.59 ± 1.58%, outperforming ethyl acetate and aqueous extracts [55]. This variation in antioxidant potential could be attributed to differences in plant variety, geographical origin, and extraction protocols, all of which influence the phytochemical composition [42,56,57].

Among the bioactive constituents of garlic, certain polyphenols and flavonoids, such as trans-ferulic acid, have garnered attention for their remarkable antioxidant properties [58]. Notably, the aqueous extract stands out for its elevated concentration of trans-ferulic acid (92.18 mg/kg fw), indicative of robust antioxidant activity. The methanolic extract, particularly abundant in flavonoids, also demonstrates significant antioxidative potential. The substantial presence of cirsiliol (417.26 mg/kg fw) and apigenin-7-O-glucoside (1.11 mg/kg fw), recognized for their antioxidative properties, in the methanolic extract, underscores its potent antioxidant capacity. These results align with prior research highlighting the antioxidative potentials of *A. sativum* extracts, attributing them to the presence of phenolic and other bioactive compounds [59]. The correlation between the detected biomolecule concentrations and the antioxidant effects of different extracts was assessed using Pearson’s and Spearman’s correlation coefficients. Phenolic compounds exhibited a strong and significant positive correlation with antioxidant activity (r = 0.99, with a p-value of 0.014). Spearman Correlation Coefficient (ρ) for this compound** **= 1.0 that confirmed a perfect monotonic relationship). Similarly, water-soluble vitamins showed a strong positive correlation (r = 0.85, ρ = 1.0, ρ = 1.0), though the relationship was not statistically significant (p = 0.15).

The high concentrations of cirsiliol and trans-ferulic acid in the polar extract suggest that this extract exhibits strong antioxidant activity. It is also plausible that organosulfur compounds, known for their antioxidative effects, contribute to this activity. Unfortunately, these compounds were not quantified in the present study, which represents a limitation. Further investigation into these bioactive molecules is warranted to better elucidate their roles in the observed antioxidant activities [60].

### Antifungal effects of different *A. sativum* extracts

The antifungal activity of *A. sativum* extracts was evaluated by measuring the diameter of the inhibition zones after 48 h of incubation with fungal strains. Table 8 displays the results observed at various concentrations of the extracts. Among the tested extracts, the aqueous extract demonstrated the most pronounced antifungal activity, with inhibition zones of 2 cm for both *A. flavus* and *A. niger*, surpassing carbendazim (0.55 cm and 0.40 cm, respectively) at a concentration of 10 mg/mL. The methanolic extract also showed notable antifungal activity (1.50 cm for *A. flavus* and 1.17 cm for *A. niger*) at the same concentration, followed by the ethyl acetate extract, which showed 0.70 cm and 0.47 cm inhibition zones, respectively, for the same strains, while the hexane extract exhibited relatively smaller inhibition zones.

**Table 8:** *A. sativum* extracts antifungal activities (expressed as inhibition zone (cm)).

Extracts	Concentration (mg/mL)	*A. flavus*	*A. niger*
**Hexane extract**	2.5	0.00 ± 0.00	0.10 ± 0.13
	5	0.00 ± 0.00	0.30 ± 0.07
	10	0.30 ± 0.20	0.37 ± 0.04
	20	1.00 ± 0.07	0.43 ± 0.22
	40	1.47 ± 0.04	0.97 ± 0.22
**Ethyl acetate extract**	2.5	0.23 ± 0.31	0.00 ± 0.00
	5	0.33 ± 0.22	0.43 ± 0.09
	10	0.70 ± 0.33	0.47 ± 0.04
	20	1.70 ± 0.07	0.50 ± 0.07
	40	1.80 ± 0.13	1.17 ± 0.22
**Methanolic extract**	2.5	0.40 ± 0.07	0.00 ± 0.00
	5	1.00 ± 0.07	0.50 ± 0.33
	10	1.50 ± 0.07	1.17 ± 0.22
	20	1.50 ± 0.07	1.33 ± 0.22
	40	2.00 ± 0.07	1.50 ± 0.07
**Aqueous extract**	2.5	1.17 ± 0.22	1.67 ± 0.44
	5	2.00 ± 0.07	1.83 ± 0.22
	10	2.00 ± 0.07	2.00 ± 0.13
	20	2.00 ± 0.13	2.00 ± 0.13
	40	2.00 ± 0.067	2.00 ± 0.13
**Carbendazim fongicide**	10	0.55 ± 0.02	0.40 ± 0.01

Values in columns are the average of three analyses ± SD.

This trend aligns with previous studies highlighting the antifungal efficacy of *A. sativum* [10,26,61–63]. Research on Pakistani *A. sativum* extracts similarly reported strong antifungal activity across various solvents, including aqueous, acetone, ethanol, and methanol, with inhibition zones ranging from 7.4–10.3 mm for *A. flavus* and 9–12.5 mm for *A. niger* [64]. Additionally, Ashraf *et al.* demonstrated the significant antifungal potential of garlic essential oil, achieving maximum inhibition zones of 18.3 ± 0.29 mm for *A. flavus* and 16.1 ± 0.43 mm for *A. niger* after three days of treatment with 100 μL of essential oil [65].

The minimum inhibitory concentration (MIC), of each garlic extract was tested, and results were presented in Table 9. The aqueous extract exhibited the most potent inhibition of fungal growth, with MIC values of 1.5 mg/mL for *A. flavus*, and 3 mg/mL for *A. niger*. The methanolic extract also exhibited significant efficacy, with MIC values of 5 mg/mL and 10 mg/mL for the respective strains. In contrast, the ethyl acetate and the hexane extracts demonstrated weaker inhibitory fungal activities with MIC values of 10 mg/mL and 20 mg/mL, respectively.

**Table 9:** Minimum inhibitory concentration (mg/mL) of tested *A. sativum* extracts.

Tested fungi	*A. flavus*	*A. niger*
Aqueous extract	1.5	3
Methanolic extract	5	10
Ethyl acetate extract	10	10
Hexane extract	20	20

Our findings reveal a possible close relationship between the polyphenol content and the observed antifungal activity. Specifically, the aqueous extract emerged as the richest in polyphenols and displayed the strongest antifungal activity. The correlation analysis showed that phenolic acids had a strong and significant positive correlation with antifungal activities against *A. flavus* (Pearson’s r = 0.973, p = 0.027) and *A. niger* (Pearson’s r = 0.999, p = 0.001). Water-soluble vitamins also exhibited strong correlations, particularly with Spearman’s ρ = 1.0 for both fungi. In contrast, flavonoids displayed weak and non-significant correlations.

These findings highlight the significant antifungal potential of the aqueous and methanolic extracts, which are richer in polyphenols. However, the antifungal efficacy was not strictly proportional to the polyphenol content, suggesting the involvement of other bioactive compounds. This may be attributed to organosulfur compounds, widely recognized for their potent antifungal properties [10] but not quantified in this study. For instance, previous research demonstrated a correlation between higher allicin content and enhanced antifungal activity in water extracts compared to ethanol extracts [42]. This limitation underscores the need for future investigations to identify and characterize these compounds in the extracts.

### Cytotoxic effects of garlic extracts

**Fig 1:**
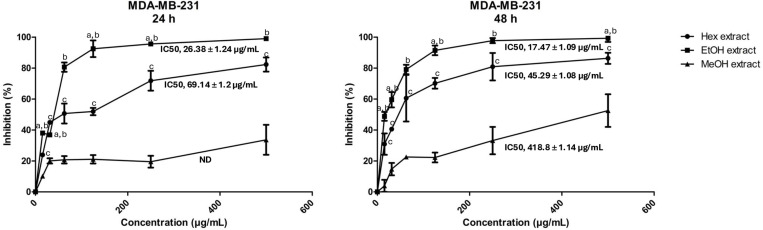
Cytotoxic effect of *A. sativum* extracts on *MDA-MB-231* cells. The MTT assay was conducted to assess cytotoxicity *in vitro* against *MDA-MB-231* cells*.* Dose-response curves were generated to evaluate the inhibition of proliferation after 24 h and 48 h of treatment with escalating concentrations (15.56, 31.125, 62.25, 125, 250, and 500 μg/mL) of different solvent extracts: Hex (hexane), EtOH (ethyl acetate), and MeOH (methanol). Three independent experiments were performed in triplicate. All values are expressed as mean ± SD (n = 3) and are presented as a percentage relative to the vehicle control. The IC50 for the 24 h exposure to the methanol extract was not determined due to an incomplete curve. All concentration points were statistically significant compared to the negative control (unless P < 0.05), except for the first point (15.56 μg/mL) of the methanol extract after 48 h of treatment. Statistical comparisons were as follows: a, P < 0.05 ethyl acetate vs. hexane; b, P < 0.05 ethyl acetate vs. methanol; and c, P < 0.05 methanol vs. hexane.

The cytotoxic effect of *A. sativum* extracts was assessed using the MTT assay following 24 and 48 h of treatment of *U266* (refractory multiple myeloma) and *MDA-MB-231* (triple-negative breast cancer) cells with varying concentrations (15.56 μg/mL - 500 μg/mL). The results allowed the determination of IC_50_ values, representing the concentration required to achieve 50% inhibition of cell viability. Among the extracts tested, methanolic extract demonstrated moderate cytotoxicity, with maximum inhibition not exceeding 55% at the highest concentration (500 μg/mL) for both cell lines (Fig 1). The IC50 value for the methanolic extract after 48 h of treatment on *MDA-MB-231* cells was estimated at approximately 418.8 ± 1.14 μg/mL. Interestingly, significant differences in cytotoxic effects were observed between extraction methods. The hexane extract exhibited greater potency, with IC50 values of 69.14 ± 1.2 μg/mL and 45.29 ± 1.08 μg/mL after 24 and 48 h, respectively. Ethyl acetate extract demonstrated the highest efficacy against the metastatic breast cancer cell line, with IC50 values of 26.38 ± 1.24 μg/mL and 17.47 ± 1.09 μg/mL after 24 and 48 hours of treatment, respectively (Fig 1).

Previous studies have shown a correlation between the cytotoxic activity of garlic extracts and their composition [66]. However, few studies have explored the impact of different extraction solvents on cytotoxicity [67]. Recent literature has associated garlic consumption with a reduced risk of breast cancer, while garlic-derived compounds have demonstrated inhibitory effects on breast cancer cells [68,69]. Notably, Isbilen and Volkan demonstrated that ethanolic *A. sativum* extract exhibited an inhibitory effect against *MDA-MB-231* cells, with an IC50 value of 2874.2 μg/mL after 48 h of treatment [70]. Comparatively, the IC50 value observed in this study for methanolic extract (418.8 μg/mL) suggests greater efficacy of our extract. The most striking finding of this work, however, lies in the exceptional potency of the ethyl acetate extract, which achieved significantly lower IC50 values compared to other extracts. This highlights the potential superiority of ethyl acetate as a solvent for isolating bioactive compounds, such as lipophilic or sulfur-containing compounds, known for their anticancer properties, although these specific compounds were not quantified in our study. For instance, the works of Malki et al. and Na et al., highlight the promising inhibitory effect of diallyl trisulfide (DATS) on the MCF-7 human breast cancer cell line through an apoptotic death mechanism [71,72]. Similarly, Sato’s recent work confirmed the potential of diallyl disulfide (DADS) and DATS as promising agents for preventing and treating breast cancer by decreasing STS protein expression and suppressing active estrogen levels in MCF-7 cells [73]. Interestingly, Marni et al. demonstrated that DADS and DATS exhibited high cytotoxicity against two paclitaxel-resistant triple-negative breast cancer cell lines, *MDA-MB-231* PR and MDA-MB-468 PR, by blocking the cell cycle [69]. These findings underscore the potential of garlic-derived compounds in breast cancer treatment and highlight the need to further explore ethyl acetate as a solvent for isolating active compounds with enhanced cytotoxic activity.

**Fig 2:**
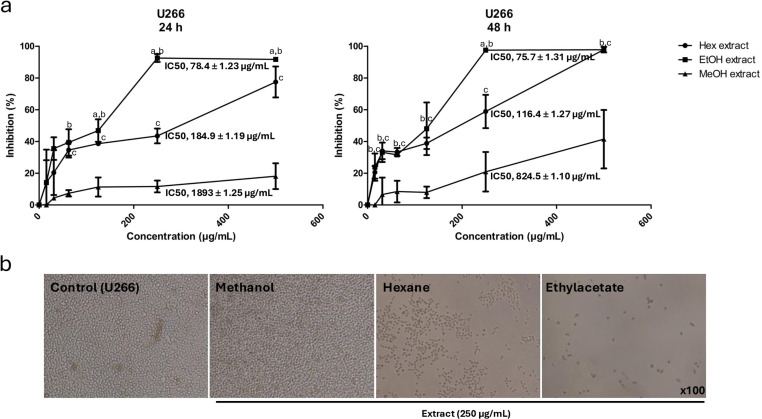
Effect of *A. sativum* extracts on *U266* multiple myeloma cells. (a) The cytotoxic effects of different solvent extracts Hex (hexane), EtOAc (ethyl acetate), and MeOH (methanol)—were evaluated after 24 h and 48 h of treatment with escalating concentrations (15.56–500 μg/mL). Values are expressed as mean ± SD (n = 3) from three independent assays and presented as percentages relative to the vehicle control. All concentration points were statistically significant compared to the negative control (P < 0.05), except for the first point (15.56 μg/mL) of the hexane and methanol extracts, and the second point of the methanol extract after 48 h of treatment. Statistical comparisons are indicated as follows: a, P < 0.05 ethyl acetate vs. hexane; b, P < 0.05 ethyl acetate vs. methanol; and c, P < 0.05 methanol vs. hexane. (b) Representative phase-contrast inverted microscopy images of *U266* cells treated or untreated (vehicle control) with 250 μg/mL of the different extracts (magnification, × 100).

For *U266* cells, the IC50 values for the hexane extract were 184.9 ± 1.19 μg/mL and 116.4 ± 1.27 μg/mL after 24 and 48 h, respectively. Remarkably, when extraction was performed in ethyl acetate, the cytotoxic effect on refractory myeloma cells was higher (Fig 2a) with IC50 values at 24 and 48 h of 78.4 ± 1.23 μg/mL and 75.7 ± 1.31 μg/mL, respectively. Observations from phase-contrast inverted microscopy corroborated the MTT assay results (Fig 2b), showing no visible reduction in the number of untreated *U266* cells (control) compared to those treated with methanolic extract. However, a significant decrease in cell viability was observed in cells treated with 250 µg/mL of *A. sativum* extracted in hexane and ethyl acetate, indicating a potent cytotoxic effect.

According to the literature, no studies have been found on multiple myeloma using garlic extracts, with only two studies investigating DADS and DATS. The first study suggested that co-treatment with DATS and dexamethasone inhibited the proliferation of two multiple myeloma cell lines. Last year, Wei Hu et al. demonstrated for the first time that DADS induces apoptosis in multiple myeloma cells through a mitochondria-dependent pathway and enhances the activity of two anti-myeloma drugs via synergistic effects [74]. These findings, along with ours, suggest that garlic-derived compounds or extracts could represent promising candidates for complementary approaches in multiple myeloma treatment. However, further studies are necessary to clarify the mechanisms of action and identify the specific bioactive components responsible for these effects.

The bioactivities of the extracts, including antioxidant, antifungal, and anticancer effects, are driven by the synergistic actions of molecules like polyphenols, phenolic acids, flavonoids, and vitamins. These compounds interact with cellular pathways to exert their effects. Polyphenols neutralize reactive oxygen species (ROS) and free radicals, boosting antioxidant enzymes like SOD and CAT. Vitamins C and E enhance antioxidant defenses, protecting cellular structures from oxidative damage. They also disrupt fungal cell membranes, inhibit ergosterol biosynthesis, and boost immune responses. In anticancer mechanisms, polyphenols and flavonoids regulate apoptosis, cell cycle arrest, and angiogenesis, while phenolic acids suppress tumor growth. Vitamins C and E repair oxidative DNA damage and promote cancer cell apoptosis [69,74].

## Conclusions

This study provides significant insights into the bioactive potential of Tunisian *A. sativum* extracts, emphasizing their antioxidant, antifungal, and cytotoxic activities. By employing successive solvent extraction techniques, we successfully isolated and quantified key compounds, including polyphenols and certain vitamins, revealing the variability in bioactive compound recovery based on solvent polarity. Notably, the ethyl acetate extract exhibited remarkable cytotoxic effects, particularly against metastatic breast cancer (*MDA-MB-231*) and refractory multiple myeloma (*U266*) cell lines, representing a novel and promising finding in the context of garlic research.

The chemical profile and bioactivity of Tunisian garlic reflect its unique environmental and agricultural context. This study highlights the richness of Tunisian *A. sativum* and its potential as a source of therapeutic agents, particularly in regions with limited access to conventional treatments. Despite these promising results, it is essential to acknowledge that this work serves as a foundational study. The observed activities require further investigation to identify and characterize the active compounds responsible and to elucidate their underlying mechanisms of action. Additionally, in vivo studies and optimized extraction techniques are necessary to validate the therapeutic potential of garlic extracts.

In conclusion, this study demonstrates the value of Tunisian *A. sativum* as a source of bioactive compounds, paving the way for its integration into functional foods and pharmaceutical research. By bridging traditional knowledge and scientific inquiry, this work lays the groundwork for developing innovative strategies to combat fungal infections and aggressive cancers, such as multiple myeloma, while highlighting the untapped potential of regional biodiversity.

## References

[1] Oliveira G, Volino-Souza M, Conte-Júnior CA, Alvares TS. Food-derived polyphenol compounds and cardiovascular health: a nano-technological perspective. Food Biosci. 2021;41:101033. doi: 10.1016/j.fbio.2021.101033

[2] Dolecek TA, McCarthy BJ, Joslin CE, Peterson CE, Kim S, Freels SA, et al. Prediagnosis food patterns are associated with length of survival from epithelial ovarian cancer. J Am Diet Assoc. 2010;110(3):369–82. doi: 10.1016/j.jada.2009.11.014 20184987

[3] O’Connor EA, Evans CV, Ivlev I, Rushkin MC, Thomas RG, Martin A, et al. Vitamin and mineral supplements for the primary prevention of cardiovascular disease and cancer: updated evidence report and systematic review for the US Preventive Services Task Force. JAMA. 2022;327(23):2334–47. doi: 10.1001/jama.2021.15650 35727272 PMC13207626

[4] Venturelli S, Leischner C, Helling T, Burkard M, Marongiu L. Vitamins as possible cancer biomarkers: significance and limitations. Nutrients. 2021;13(11):3914. doi: 10.3390/nu13113914 34836171 PMC8622959

[5] Limam I, Ben Aissa-Fennira F, Essid R, Chahbi A, Kefi S, Mkadmini K, et al. Hydromethanolic root and aerial part extracts from Echium arenarium Guss suppress proliferation and induce apoptosis of multiple myeloma cells through mitochondrial pathway. Environ Toxicol. 2021;36(5):874–86. doi: 10.1002/tox.23090 33393729

[6] Limam I, Abdelkarim M, Essid R, Chahbi A, Fathallah M, Elkahoui S, et al. Olea europaea L. cv. Chetoui leaf and stem hydromethanolic extracts suppress proliferation and promote apoptosis via caspase signaling on human multiple myeloma cells. Eur J Integr Med. 2020;37:101145. doi: 10.1016/j.eujim.2020.101145

[7] Kharrat R, Lakhal FB, Souia H, Limam I, Naji HB, Abdelkarim M. Anticancer effects of Artemisia campestris extract on acute myeloid leukemia cells: an ex vivo study. Med Oncol. 2024;41(8):206. doi: 10.1007/s12032-024-02453-y 39037595

[8] Asemani Y, Zamani N, Bayat M, Amirghofran Z. Allium vegetables for possible future of cancer treatment. Phytother Res. 2019;33(12):3019–39. doi: 10.1002/ptr.6490 31464060

[9] Li Q-Q, Zhou S-D, He X-J, Yu Y, Zhang Y-C, Wei X-Q. Phylogeny and biogeography of *Allium* (Amaryllidaceae: Allieae) based on nuclear ribosomal internal transcribed spacer and chloroplast rps16 sequences, focusing on the inclusion of species endemic to China. Ann Bot. 2010;106(5):709–33. doi: 10.1093/aob/mcq177 20966186 PMC2958792

[10] Okoro BC, Dokunmu TM, Okafor E, Sokoya IA, Israel EN, Olusegun DO, et al. The ethnobotanical, bioactive compounds, pharmacological activities and toxicological evaluation of garlic (*Allium sativum*): a review. Pharmacol Res - Mod Chin Med. 2023;8:100273. doi: 10.1016/j.prmcm.2023.100273

[11] Choudhary S, Noor MU, Hussain MS. Pharmacological properties and phytoconstituents of garlic (Allium sativum L.): A review. Biol Sci. 2022;02(04). doi: 10.55006/biolsciences.2022.2402

[12] Le Bon A-M. Alliacées et prévention des cancers. Phytothérapie. 2016;14(3):159–64. doi: 10.1007/s10298-016-1041-8

[13] Misiorek M, Sekuła J, Ruman T. Mass spectrometry imaging of low molecular weight compounds in garlic (*Allium sativum* L.) with gold nanoparticle enhanced target: Mass spectrometry imaging of *Allium sativum* L. Phytochem Anal. 2017;28(6):479–86. doi: 10.1002/pca.2696 28612465

[14] Peterssen-Fonseca D, Henríquez-Aedo K, Carrasco-Sandoval J, Cañumir-Veas J, Herrero M, Aranda M. Chemometric optimisation of pressurised liquid extraction for the determination of alliin and S-allyl-cysteine in giant garlic (*Allium ampeloprasum* L.) by liquid chromatography tandem mass spectrometry. Phytochem Anal. 2021;32(6):1051–8. doi: 10.1002/pca.3046 33779027

[15] Jikah AN, Edo GI, Makia RS, Yousif E, Gaaz TS, Isoje EF, et al. A review of the therapeutic potential of sulfur compounds in *Allium sativum*. Measurement: Food. 2024;15:100195. doi: 10.1016/j.meafoo.2024.100195

[16] Choi Y-J, Lim J-Y, Kang M-J, Choi J-Y, Yang J-H, Chung YB, et al. Changes in bacterial composition and metabolite profiles during kimchi fermentation with different garlic varieties. Heliyon. 2024;10(2):e24283. doi: 10.1016/j.heliyon.2024.e24283 38293374 PMC10826663

[17] El-Saber Batiha G, Magdy Beshbishy A, G Wasef L, Elewa YHA, A Al-Sagan A, Abd El-Hack ME, et al. Chemical constituents and pharmacological activities of garlic (*Allium sativum* L.): A Review. Nutrients. 2020;12(3):872. doi: 10.3390/nu12030872 32213941 PMC7146530

[18] Rahman K, Lowe GM, Smith S. Aged garlic extract inhibits human platelet aggregation by altering intracellular signaling and platelet shape change. J Nutr. 2016;146(2):410S-415S. doi: 10.3945/jn.114.202408 26764324

[19] Lachovicz R, Ferro-Lebres V, Almeida-de-Souza J. Phytotherapy: a systematic review for the treatment of hypertension. J Herb Med. 2025;50:100985. doi: 10.1016/j.hermed.2024.100985

[20] Naji KM, Al-Shaibani ES, Alhadi FA, Al-Soudi SA, D’souza MR. Hepatoprotective and antioxidant effects of single clove garlic against CCl4-induced hepatic damage in rabbits. BMC Complement Altern Med. 2017;17(1):411. doi: 10.1186/s12906-017-1916-8 28818066 PMC5561638

[21] Subroto E, Cahyana Y, Tensiska, Mahani, Filianty F, Lembong E, et al. Bioactive compounds in garlic (*Allium sativum* L.) as a source of antioxidants and its potential to improve the immune system: a review. Food Res. 2021;5(6):1–11. doi: 10.26656/fr.2017.5(6).042

[22] Chekki RZ, Snoussi A, Hamrouni I, Bouzouita N. Chemical composition, antibacterial and antioxidant activities of Tunisian garlic (*Allium sativum*) essential oil and ethanol extract. Mediterr J Chem. 2014;3(4):947–56. doi: 10.13171/mjc.3.4.2014.09.07.11

[23] Nishimura H, Higuchi O, Tateshita K. Antioxidative activity of sulfur-containing compounds in *Allium* species for human LDL oxidation *in vitro*. Biofactors. 2004;21(1–4):277–80. doi: 10.1002/biof.552210154 15630211

[24] Capasso A. Antioxidant action and therapeutic efficacy of *Allium sativum* L. Molecules. 2013;18(1):690–700. doi: 10.3390/molecules18010690 23292331 PMC6269925

[25] Hayat S, Ahmad A, Ahmad H, Hayat K, Khan MA, Runan T. Garlic, from medicinal herb to possible plant bioprotectant: a review. Sci Hortic. 2022;304:111296. doi: 10.1016/j.scienta.2022.111296

[26] Shang A, Cao S-Y, Xu X-Y, Gan R-Y, Tang G-Y, Corke H, et al. Bioactive compounds and biological functions of garlic (*Allium sativum* L.). Foods. 2019;8(7):246. doi: 10.3390/foods8070246 31284512 PMC6678835

[27] Thomson M, Ali M. Garlic [*Allium sativum*]: a review of its potential use as an anti-cancer agent. Curr Cancer Drug Targets. 2003;3(1):67–81. doi: 10.2174/1568009033333736 12570662

[28] Ayed C, Mezghani N, Rhimi A, AL Mohandes Dridi B. Morphological evaluation of Tunisian garlic (*Allium sativum* L.) landraces for growth and yield traits. J Hortic Postharvest Res. 2019;2:43–52. doi: 10.22077/jhpr.2018.1838.1033

[29] Institute IPGR. IPGRI Annual Report 2002. 2003. Available from: https://hdl.handle.net/10568/97486

[30] Shetty S, Thomas B, Shetty V, Bhandary R, Shetty RM. An in-vitro evaluation of the efficacy of garlic extract as an antimicrobial agent on periodontal pathogens: a microbiological study. Ayu. 2013;34(4):445–51. doi: 10.4103/0974-8520.127732 24695825 PMC3968712

[31] Jaouadi O, Limam I, Abdelkarim M, Berred E, Chahbi A, Caillot M, et al. 5,6-epoxycholesterol isomers induce oxiapoptophagy in myeloma cells. Cancers (Basel). 2021;13(15):3747. doi: 10.3390/cancers13153747 34359648 PMC8345143

[32] Limam I, Abdelkarim M, El Ayeb M, Crepin M, Marrakchi N, Di Benedetto M. Disintegrin-like protein strategy to inhibit aggressive triple-negative breast cancer. Int J Mol Sci. 2023;24(15):12219. doi: 10.3390/ijms241512219 37569595 PMC10418936

[33] Limam I, Ghali R, Abdelkarim M, Ouni A, Araoud M, Abdelkarim M, et al. Tunisian Artemisia campestris L.: a potential therapeutic agent against myeloma - phytochemical and pharmacological insights. Plant Methods. 2024;20(1). doi: 10.1186/s13007-024-01185-4 PMC11067135 38698384

[34] Kirby AJ, Schmidt RJ. The antioxidant activity of Chinese herbs for eczema and of placebo herbs--I. J Ethnopharmacol. 1997;56(2):103–8. doi: 10.1016/s0378-8741(97)01510-9 9174970

[35] Jorgensen JH, Turnidge JD. Susceptibility test methods: dilution and disk diffusion methods. Manual of Clinical Microbiology. John Wiley & Sons, Ltd. 2015. pp. 1253–73. doi: 10.1128/9781555817381.ch71

[36] Arendrup MC, Meletiadis J, Mouton JW, Guinea J, Cuenca-Estrella M, Lagrou K, et al. EUCAST technical note on isavuconazole breakpoints for Aspergillus, itraconazole breakpoints for Candida and updates for the antifungal susceptibility testing method documents. Clin Microbiol Infect. 2016;22(6):571.e1–4. doi: 10.1016/j.cmi.2016.01.017 26851656

[37] Wiegand I, Hilpert K, Hancock REW. Agar and broth dilution methods to determine the minimal inhibitory concentration (MIC) of antimicrobial substances. Nat Protoc. 2008;3(2):163–75. doi: 10.1038/nprot.2007.521 18274517

[38] Abdelkarim M, Ben Younes K, Limam I, Guermazi R, ElGaaied ABA, Aissa-Fennira FB. 3,6-dichloro-1,2,4,5-tetrazine assayed at high doses in the metastatic breast cancer cell line MDA-MB-231 reduces cell numbers and induces apoptosis. Curr Bioact Compd. 2020;16(4):546–50. doi: 10.2174/1573407215666181224105826

[39] Kim K-H, Park JK, Choi Y-W, Kim Y-H, Lee EN, Lee J-R, et al. Hexane extract of aged black garlic reduces cell proliferation and attenuates the expression of ICAM-1 and VCAM‑1 in TNF-α-activated human endometrial stromal cells. Int J Mol Med. 2013;32(1):67–78. doi: 10.3892/ijmm.2013.1362 23619991

[40] Cavalcanti VP, Aazza S, Bertolucci SKV, Rocha JPM, Coelho AD, Oliveira AJM, et al. Solvent mixture optimization in the extraction of bioactive compounds and antioxidant activities from garlic (*Allium sativum* L.). Molecules. 2021;26(19):6026. doi: 10.3390/molecules26196026 34641570 PMC8512559

[41] Carreón-Delgado DF, Hernández-Montesinos IY, Rivera-Hernández KN, Del Sugeyrol Villa-Ramírez M, Ochoa-Velasco CE, Ramírez-López C. Evaluation of pretreatments and extraction conditions on the antifungal and antioxidant effects of garlic (*Allium sativum*) peel extracts. Plants (Basel). 2023;12(1):217. doi: 10.3390/plants12010217 36616344 PMC9823915

[42] Bar M, Binduga UE, Szychowski KA. Methods of isolation of active substances from garlic (*Allium sativum* L.) and its impact on the composition and biological properties of garlic extracts. Antioxidants (Basel). 2022;11(7):1345. doi: 10.3390/antiox11071345 35883836 PMC9312217

[43] Elhafez ZAA. Influence of some solvents on the extraction of major phenolic compounds and their antioxidant activities in Egyptian Garlic (*Allium sativum* L.). Asian J Agric Hortic Res. 2021;:99–106. doi: 10.9734/ajahr/2021/v8i430149

[44] Nagella P, Thiruvengadam M, Ahmad A, Yoon J-Y, Chung I-M. Composition of polyphenols and antioxidant activity of garlic bulbs collected from different locations of Korea. Asian J Chem. 2014;26(3):897–902. doi: 10.14233/ajchem.2014.16143a

[45] Chahbani A, Benissa Z, Jridi M, Zouari N, Fakhfakh N. Thermal processing impacts on drying kinetics, LC–ESI–MS based chemical profiling, and antioxidant capacity of garlic (*Allium sativum* L.) Leaves. Chem Afr. 2024;7(8):4325–35. doi: 10.1007/s42250-024-01055-5

[46] Szychowski K, Rybczyńska-Tkaczyk K, Gaweł-Bęben K, Świeca M, Karaś M, Jakubczyk A, et al. Characterization of active compounds of different garlic (*Allium sativum* L.) cultivars. Pol J Food Nutr Sci. 2018;68(1):73–81. doi: 10.1515/pjfns-2017-0005

[47] Beato VM, Orgaz F, Mansilla F, Montaño A. Changes in phenolic compounds in garlic (*Allium sativum* L.) owing to the cultivar and location of growth. Plant Foods Hum Nutr. 2011;66(3):218–23. doi: 10.1007/s11130-011-0236-2 21667145

[48] Ayyanar M, Krupa J, Jenipher C, Amalraj S, Gurav SS. Phytochemical composition, in vitro antioxidant and antibacterial activity of Moringa concanensis Nimmo leaves. Vegetos. 2023;37(4):1377–88. doi: 10.1007/s42535-023-00663-9

[49] Natália Č, Judita L, Eduard P, Marek Š, Hana F, Monika Ň, et al. Total polyphenol content, total flavonoid content, and antioxidant activity of garlic (*Allium sativum* L.) cultivars. J Microb Biotech Food Sci. 2023;:e9668. doi: 10.55251/jmbfs.9668

[50] Utama GL, Rahmi Z, Sari MP, Hanidah I-I. Psychochemical changes and functional properties of organosulfur and polysaccharide compounds of black garlic (*Allium sativum* L.). Curr Res Food Sci. 2024;8:100717. doi: 10.1016/j.crfs.2024.100717 38559380 PMC10978486

[51] Gambelli L, Marconi S, Durazzo A, Camilli E, Aguzzi A, Gabrielli P, et al. Vitamins and minerals in four traditional garlic ecotypes (*Allium sativum* L.) from Italy: an example of territorial biodiversity. Sustainability. 2021;13(13):7405. doi: 10.3390/su13137405

[52] Wang Y, Tong L, Yang L, Ren B, Guo D. Metabolite profiling and antioxidant capacity of natural Ophiocordyceps gracilis and its cultures using LC-MS/MS-based metabolomics: comparison with Ophiocordyceps sinensis. Phytochem Anal. 2024;35(2):308–20. doi: 10.1002/pca.3289 37779226

[53] Dalhat MH, Adefolake FA, Musa M. nutritional composition and phytochemical analysis of aqueous extract of *Allium cepa* (Onion) and *Allium sativum* (Garlic). Asian Food Sci J. 2018;3(4):1–9. doi: 10.9734/afsj/2018/43165

[54] Wani S, Basir SF. Analysis of antioxidant activity, total phenolic and total flavonoid contents of *Allium sativum*, Mentha arvensis and Murraya koenigii. Int J Adv Sci Res Eng. 2018;7:2632–46.

[55] Awan KA, Butt MS, Haq IU, Suleria HAR. Investigating the Antioxidant Potential of Garlic (*Allium sativum*) Extracts Through Different Extraction Modes. [cited 7 Jan 2025] Available from: https://www.eurekaselect.com/article/86497

[56] Sam Arul Raj M, Amalraj S, Alarifi S, Kalaskar MG, Chikhale R, Santhi VP, et al. Nutritional composition, mineral profiling, in vitro antioxidant, antibacterial and enzyme inhibitory properties of selected Indian Guava cultivars leaf extract. Pharmaceuticals (Basel). 2023;16(12):1636. doi: 10.3390/ph16121636 38139763 PMC10747950

[57] Divya M, Shanti G, Amalraj S, Amiri-Ardekani E, Gurav S, Ayyanar M. Evaluation of in vitro enzyme inhibitory, anti-inflammatory, antioxidant, and antibacterial activities of *Oldenlandia corymbosa* L. and *Oldenlandia umbellata* L. whole plant extracts. Pharmacol Res - Mod Chin Med. 2023;8:100286. doi: 10.1016/j.prmcm.2023.100286

[58] Marcato DC, Spagnol CM, Salgado HRN, Isaac VLB, Corrêa MA. New and potential properties, characteristics, and analytical methods of ferulic acid: a review. Braz J Pharm Sci. 2022;58. doi: 10.1590/s2175-97902020000118747

[59] Güçlü E, Çinar Ayan İ. Apigenin-7-glucoside attenuates hydrogen peroxide-induced oxidative stress and neuronal death in SH-SY5Y cells via activation of antioxidant enzymes system and inhibition of caspases genes expression. Genel Tıp Dergisi. 2023;33(2):162–8. doi: 10.54005/geneltip.1219084

[60] Jang H-J, Lee H-J, Yoon D-K, Ji D-S, Kim J-H, Lee C-H. Antioxidant and antimicrobial activities of fresh garlic and aged garlic by-products extracted with different solvents. Food Sci Biotechnol. 2017;27(1):219–25. doi: 10.1007/s10068-017-0246-4 30263743 PMC6049750

[61] Fratianni F, Riccardi R, Spigno P, Ombra MN, Cozzolino A, Tremonte P, et al. Biochemical characterization and antimicrobial and antifungal activity of two endemic varieties of garlic (*Allium sativum* L.) of the Campania Region, Southern Italy. J Med Food. 2016;19(7):686–91. doi: 10.1089/jmf.2016.0027 27259073

[62] Carreón-Delgado DF, Hernández-Montesinos IY, Rivera-Hernández KN, Del Sugeyrol Villa-Ramírez M, Ochoa-Velasco CE, Ramírez-López C. Evaluation of pretreatments and extraction conditions on the antifungal and antioxidant effects of garlic (*Allium sativum*) Peel Extracts. Plants (Basel). 2023;12(1):217. doi: 10.3390/plants12010217 36616344 PMC9823915

[63] Daniel D, Haruna A, Musa ABK . Antifungal activity of garlic (*Allium sativum*) extract on some selected fungi. J Med Herb Ethnomedicine. 1970;:12–4. doi: 10.25081/jmhe.2018.v4.3383

[64] Abdul Qadir M, Shahzadi SK, Bashir A, Munir A, Shahzad S. Evaluation of phenolic compounds and antioxidant and antimicrobial activities of some common herbs. Int J Anal Chem. 2017;2017:3475738. doi: 10.1155/2017/3475738 28316626 PMC5337800

[65] Ashraf SA, Ahmad Khan M, Awadelkareem AM, Tajuddin S, Ahmad MF, Hussain T. GC-MS analysis of commercially available *Allium sativum* and *Trigonella foenum-graecum* essential oils and their antimicrobial activities. J Pure Appl Microbiol. 2019;13(4):2545–52. doi: 10.22207/jpam.13.4.69

[66] Țigu AB, Moldovan CS, Toma V-A, Farcaș AD, Moț AC, Jurj A, et al. Phytochemical Analysis and in vitro effects of *Allium fistulosum* L. and *Allium sativum* L. extracts on human normal and tumor cell lines: a comparative study. Molecules. 2021;26(3):574. doi: 10.3390/molecules26030574 33499159 PMC7866094

[67] Furdak P, Pieńkowska N, Kapusta I, Bartosz G, Sadowska-Bartosz I. Comparison of Antioxidant and antiproliferative effects of various forms of garlic and ramsons. Molecules. 2023;28(18):6512. doi: 10.3390/molecules28186512 37764288 PMC10538172

[68] Growth inhibitory effects of diallyl disulfide on human breast cancer cell lines | Carcinogenesis | Oxford Academic. [cited 18 Feb 2024]. Available from: https://academic.oup.com/carcin/article/22/6/891/2733833 10.1093/carcin/22.6.891 11375895

[69] Marni R, Kundrapu DB, Chakraborti A, Malla R. Insight into drug sensitizing effect of diallyl disulfide and diallyl trisulfide from *Allium sativum* L. on paclitaxel-resistant triple-negative breast cancer cells. J Ethnopharmacol. 2022;296:115452. doi: 10.1016/j.jep.2022.115452 35690339

[70] Isbilen O, Volkan E. Anticancer activities of *Allium sativum* L. against MCF-7 and MDA-MB-231 breast cancer cell lines mediated by caspase-3 and caspase-9. Cyprus J Med Sci. 2021;5(4):305–12. doi: 10.5152/cjms.2020.1848

[71] Malki A, El-Saadani M, Sultan AS. Garlic constituent diallyl trisulfide induced apoptosis in MCF7 human breast cancer cells. Cancer Biol Ther. 2009;8(22):2175–85. doi: 10.4161/cbt.8.22.9882 19823037

[72] Na H-K, Kim E-H, Choi M-A, Park J-M, Kim D-H, Surh Y-J. Diallyl trisulfide induces apoptosis in human breast cancer cells through ROS-mediated activation of JNK and AP-1. Biochem Pharmacol. 2012;84(10):1241–50. doi: 10.1016/j.bcp.2012.08.024 22981381

[73] Sato A, Yabuki A, Sato G, Nemoto H, Ogawa Y, Ohira M. Garlic (*Allium sativum* L.) organosulfur compounds inhibit breast cancer cell proliferation by decreasing steroid sulfatase levels. Anticancer Res. 2025;45(1):145–52. doi: 10.21873/anticanres.17401 39740834

[74] Hu W, Sun J, Zhang Y, Chen T, He F, Zhao H, et al. Diallyl disulfide synergizes with melphalan to increase apoptosis and DNA damage through elevation of reactive oxygen species in multiple myeloma cells. Ann Hematol. 2024;103(4):1293–303. doi: 10.1007/s00277-023-05592-w 38148345

